# Evaluation of a minimal array of *Treponema pallidum* antigens as biomarkers for syphilis diagnosis, infection staging, and response to treatment

**DOI:** 10.1128/spectrum.03466-23

**Published:** 2023-12-14

**Authors:** Austin M. Haynes, Kelika A. Konda, Emily Romeis, Janet Siebert, Silver K. Vargas, Michael Reyes Diaz, Amber Phan, Carlos F. Caceres, Lorenzo Giacani, Jeffrey D. Klausner

**Affiliations:** 1 Department of Medicine, Division of Allergy and Infectious Diseases, Harborview Medical Center, University of Washington, Seattle, Washington, USA; 2 Division of Infectious Disease, David Geffen School of Medicine, University of California Los Angeles, Los Angeles, California, USA; 3 Center for Interdisciplinary Studies in Sexuality, AIDS and Society, Universidad Peruana Cayetano Heredia, Lima, Peru; 4 CytoAnalytics, Denver, Colorado, USA; 5 Department of Global Health, Harborview Medical Center, University of Washington, Seattle, Washington, USA; 6 Keck School of Medicine, University of Southern California, Los Angeles, California, USA; Ann & Robert H. Lurie Children's Hospital of Chicago, Chicago, Illinois, USA

**Keywords:** syphilis, *Treponema pallidum*, protein array, serodiagnosis, staging, treatment response

## Abstract

Syphilis is a multistage chronic sexually transmitted infection caused by the spirochete *Treponema pallidum* subsp. *pallidum* (*T. pallidum*). Today, syphilis is still endemic in low- and middle-income nations and has become resurgent in many high-income countries. Infection can lead to severe sequelae in untreated patients, while vertical transmission of *T. pallidum* is associated with high stillbirth rates and neonatal death. The combination of non-treponemal and treponemal tests allows syphilis serodiagnosis and monitoring of treatment response, albeit not without limitations. In this work, we developed a minimal array for multiple parallel detection of antibodies specific to the immunodominant *T. pallidum* antigens Tp0435 and Tp0574 and 14 additional proteins known to be immunogenic during infection but only partially or not at all evaluated for their diagnostic potential. To assess whether reactivity to these antigens could improve early syphilis diagnosis, serve as biomarkers for disease staging, and monitor response to treatment, we tested the array using 217 serum specimens longitudinally collected pre- and post-treatment from 120 syphilis patients. Results showed significantly different reactivity to a subset of antigens in pre-treatment sera (baseline) when covariates such as syphilis stage, syphilis history, and HIV status were factored in. Furthermore, reactivity to several antigens significantly decreased in post-treatment sera compared to baseline. Although studies with larger sample panels will be needed to validate these findings, this study supports the screening of *T. pallidum* proteomic arrays to identify antigens that could serve as predictors of response and antigens that could help with early syphilis diagnosis.

**IMPORTANCE:**

This manuscript explores the host humoral response to selected antigens of the syphilis agent during infection to evaluate their potential use as diagnostic tests and markers for treatment.

## INTRODUCTION

Syphilis, caused by the spirochete *Treponema pallidum* subsp. *pallidum* (*T. pallidum*), is a multistage sexually transmitted infection of significant importance for global health. The total disease burden is estimated to range from 18 to 56 million cases, while the global incidence is between 5.6 and 11 million new cases annually (1 – 5). If left untreated, syphilis can progress to affect the cardiovascular and central nervous systems, possibly leading to severe sequelae or death (6). Furthermore, 661,000 cases of congenital syphilis, associated with 200,000 stillbirths and perinatal deaths, were estimated to have occurred in 2016 (7 – 9). In the United States, after years of decline in incidence, syphilis started to undergo a significant and steady resurgence at the end of the 1990s. This new epidemic affects particularly men who have sex with men (MSM) and persons living with HIV (10), even though heterosexuals are also increasingly affected. As a result, congenital syphilis rates increased by 203% between 2017 and 2021 (11) in the United States. A resurgence in syphilis cases is also occurring in other high-income nations of North America, Europe, and Asia (12 – 15), while remaining high in low- and middle-income nations. The current syphilis burden and epidemiological trends warrant novel research endeavors to improve existing syphilis diagnostics. Such efforts, in addition to vaccine development and drug repurposing (16, 17), will be paramount in enhancing syphilis control strategies.

Primary syphilitic lesions (chancres) are generally painless and may occur on the cervix or rectum of infected individuals. As such, these lesions can be missed during routine exams. Secondary disease manifestations can also be mischaracterized. Consequently, syphilis is usually diagnosed using a combination of patient history with direct and indirect serologic tests (18). During disease latency, in the absence of clinical manifestations, serological tests are the only available option that can provide interpretable results. Direct detection through molecular approaches aimed at amplifying nucleic acids has proven very useful in complementing serology. Still, there are currently no FDA-approved molecular tests to detect *T. pallidum* in biological specimens, albeit amplification-based assays are available in CLIA-certified laboratories nationwide (19 – 21). However, molecular tests are affected by the pathogen immune-mediated partial clearance process preceding the latent stages, and although a positive pre-treatment molecular test outcome can be associated with an active infection, a negative result cannot rule infection out.

Syphilis serological tests are divided into non-treponemal tests and treponemal tests. Non-treponemal tests, such as the rapid plasma reagin (RPR) test, are flocculation assays that measure antibodies (IgM or IgG) elicited by lipoidal material released from damaged host cells and the bacterium envelope. However, because the development of these antibodies might require up to 2 weeks from the appearance of the primary lesion, about 25%–30% of primary syphilis cases may be missed by these tests (22 – 24). Without treatment, non-treponemal antibody titers generally peak 1–2 years after infection and remain positive even in late disease (25, 26). After treatment, titers decline and generally become non-reactive within 6 months in immunocompetent patients, even though in some cases, it might require up to 2 years (27). However, ~20% of syphilis-infected patients show persistent low titer of non-treponemal antibodies (serofast state) even after stage-appropriate treatment (28). A fourfold decrease in non-treponemal test titers indicates the achievement of serological cure, while a newly increased titer post-treatment is associated with reinfection. Non-treponemal tests are, therefore, instrumental in diagnosing active syphilis and assessing response to treatment (25, 26, 29). Treponemal tests such as the *T. pallidum* particle agglutination (TPPA) and *T. pallidum* hemagglutination tests use whole cell extracts to detect antibodies to *T. pallidum* antigens (29). In recent years, treponemal tests (TTs) using immunodominant *T. pallidum* antigens such as Tp0574 (TpN47), Tp0435 (TpN17), and Tp0171 (TpN15) in enzyme and chemiluminescence immunoassays have become a platform of choice in laboratories performing large-scale testing, as these assays can be automated, and the interpretation of the results is not operator-dependent (30). However, as treponemal antibodies persist over the patient’s life, treponemal tests in their current format cannot discriminate between an active and a previously treated infection. These tests become positive 6–14 days after the appearance of the chancre and, therefore, may be helpful to detect early syphilis cases undetected by non-treponemal tests but are generally used to confirm a positive non-treponemal test result, unless otherwise indicated by country-specific policies on syphilis testing algorithms (29).

Past studies focusing on treponemal tests have supported the importance of evaluating additional *T. pallidum*-specific antigens to assess their diagnostic efficiency compared to more established antigens (31, 32). These studies, however, often evaluated a relatively small number of specimens and never used convalescent sera to assess how reactivity to non-immunodominant antigens varied post-treatment. Here, we developed a 16-protein minimal array of *T. pallidum* recombinant antigens known to be immunogenic during experimental or natural syphilis infection (31) to compare the diagnostic efficiency of 14 antigens in comparison to the Tp0574 and Tp0435 immunodominant antigens. The array was tested using pre- and post-therapy sera from 122 patients with syphilis at different stages, with and without a history of previous syphilis infection, and with different HIV statuses to evaluate whether reactivity to any of these antigens could improve early diagnosis, help discriminate active versus successfully treated infections, or perform disease staging.

## RESULTS

**Fig 1:**
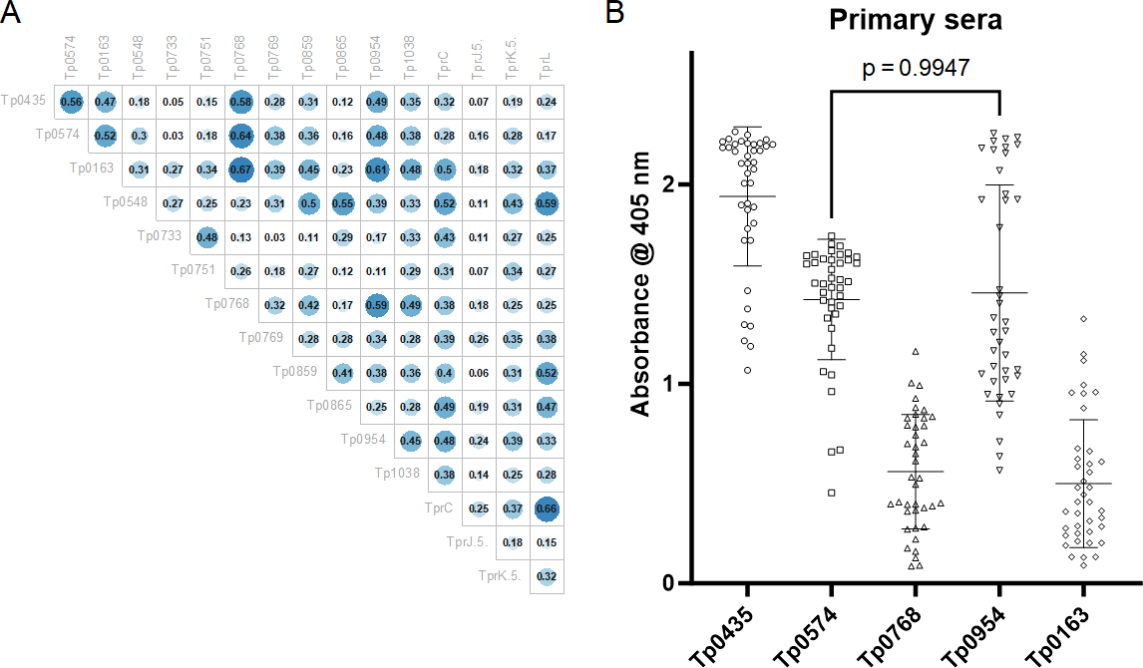
Correlation in baseline sera between immunodominant antigens and additional proteins in the array. (**A**) Pearson correlation coefficients showing that at baseline, Tp0163, Tp0768, and Tp0954 reactivity correlates with that of the immunodominant Tp0435 and Tp0574 antigens. A coefficient value ≥0.47 was used as the cut-off. (**B**) Baseline primary sera reactivity to Tp435, Tp0574, Tp0163, Tp0769, and Tp0954 plotted as individual values with mean ± standard deviation. Except for Tp0954 and Tp0574 (*P* = 0.99), the reactivity of Tp0163, Tp0954, and Tp0769 is significantly different from that of the immunodominant antigens.

We first explored whether any of the 14 additional recombinant proteins in the array could be viable options to facilitate early syphilis diagnosis. To this end, we evaluated whether the reactivity to each of the 14 antigens in the array correlated to reactivity to Tp0435 and Tp0574 in pre-treatment baseline sera by calculating Pearson correlation coefficients. For three antigens, namely, Tp0163 (the TroA/Tromp1 periplasmic component of the TroABC transporter) (33), Tp0954, a putative *T. pallidum* lipoprotein and placental adhesin (34), and Tp0768 (the TmpA lipoprotein) (35), reactivity showed a good coefficient of correlation with the two immunodominant antigens (Fig. 1A). However, in patients with primary syphilis, the mean reactivity to Tp0768 [0.52 absorbance units (AUs)] and Tp0163 (0.53 AUs) was significantly lower at baseline compared to Tp0435 (1.88 AUs) and Tp0574 (1.44 AUs). Although the mean reactivity to Tp0954 (1.47 AUs) was not significantly different from that of Tp0574 (*P* = 0.98; Fig. 1B), the values for Tp0954 were spread over a larger interval (min/max value = 0.56/2.25; SD = 0.54) compared to Tp0435 (min/max value = 0.45/1.74; SD = 0.31) and Tp0574 (min/max value = 1.06/2.26; SD = 0.30) (Fig. 1B). The descriptive statistics for baseline values of the antigens are in Table S1.

**Fig 2:**
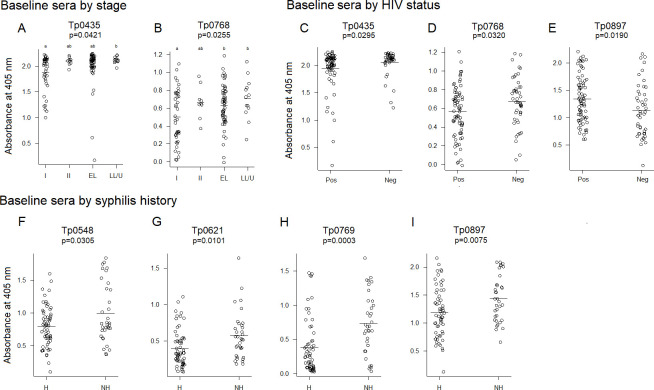
Baseline reactivity to selected *T. pallidum* antigens. Antigens to which a significant difference in reactivity was detected in baseline sera (collected at diagnosis and prior to treatment) using stage (A, B), HIV status (C–E), and syphilis history (F–I) as covariates. In panels (A and B), the *P*-value indicates that there is a significant difference across the means of the four groups analyzed. Groups lean toward statistically significant differences in means (*P* < 0.1) if they do not share a letter. I, primary syphilis; II, secondary syphilis; EL, early latent; LL, late latent; U: latency of unknown duration; Pos, HIV-positive patients; Neg: HIV-negative patients; H, patients with history of syphilis; NH, patients with no history of syphilis. The horizontal bar indicates the mean absorbance value. Analyses in panels (A, B) were performed with 118 samples. Analyses in panels (C–E) were performed with 120 samples. Analysis in panels (F–I) was performed with 93 samples.

We then investigated whether the differences in reactivity to these antigens were detectable in baseline sera once covariates were included in the model. Results showed a significant difference across the means of the four disease stages analyzed for the Tp0435 antigen (*P* = 0.0421; Fig. 2A). When stages were compared individually and results were corrected for multiple comparisons, sera from patients with primary syphilis showed significantly lower reactivity to Tp0435 than samples from late latent syphilis patients and patients in latency of unknown duration. However, no significant differences were found when primary syphilis sera were compared to either secondary or early latent sera, even though the mean absorbance values were higher in both later-stage groups compared to primary syphilis sera (Fig. 2A). A significant difference across the means of the four groups was also shown for Tp0768 (*P* = 0.0255). Also, in this case, reactivity was lower in patients with primary syphilis compared to both early latent and late latent/unknown duration sera (*P* = 0.0255) (Fig. 2B). No significant differences were, however, found when primary and secondary syphilis sera were compared to each other and when secondary syphilis sera were compared to early latent and late latent/unknown sera (Fig. 2B).

When HIV status was considered as a covariate, sera from HIV-negative patients exhibited higher reactivity to Tp0435 and Tp0768 (*P* = 0.0295 and 0.0320, respectively) compared to HIV-positive sera but lower reactivity to Tp0897/TprK.5′ (*P* = 0.0190) (Fig. 2C through E). Lastly, when a history of infection was factored in, a significantly higher reactivity to four antigens, Tp0548 (*P* = 0.0305), Tp0769 (*P* = 0.0101), Tp0897/TprK.5′ (*P* = 0.0003), and Tp0621/TprJ.5′ (*P* = 0.0075), was found in samples from patients at their first episode of syphilis (Fig. 2F through I) compared to those with a history of past syphilis infection.

**Fig 3:**
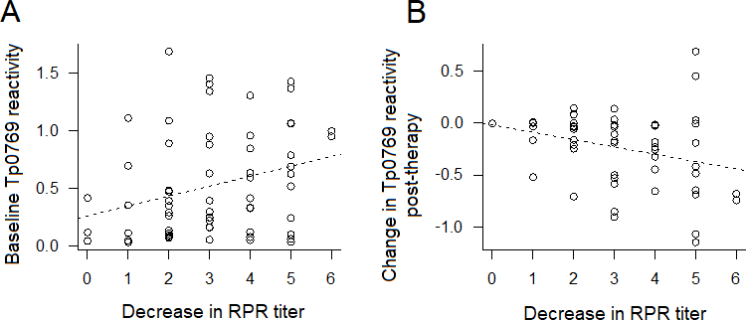
Correlation between reactivity of Tp0769 at baseline and decrease in RPR titer and between changes in Tp0769 at time point 2 (tp2) post-treatment and decrease in RPR titer. (**A**) Higher baseline levels of Tp0769 are associated with a higher RPR titer. Correlation *P*-value =0.02. (**B**) Decrease in Tp0769 post-treatment is associated with the decrease of RPR titer. Dotted lines represent a fitted regression model (antigen~decrease in RPR dilution). *N* at baseline = 64; *N* with change = 51. For the RPR titers, 0–6 values represent 1:1–1:64.

We also assessed whether a decrease in reactivity to the array antigens would occur post-treatment. To this end, we first found that reactivity to several antigens in baseline sera significantly correlated with RPR titers (*P* < 0.05) (Table 1), with Pearson correlation coefficients ≥0.47 for the Tp0769 (the TmpB lipoprotein) and Tp0859 (Table 1). However, the correlation between baseline antigen reactivity and a decrease in RPR titer at time point 2 (3 months post-treatment) was significant only for the Tp0769 (*P* = 0.02, correlation coefficient = 0.28; Fig. 3A). The correlation between the decrease in Tp0769 reactivity and the decrease in RPR titer was also significant (Fig. 3B).

**TABLE 1:** Correlation of antigen reactivity at baseline with RPR titers

Antigen	Pearson coefficient	Significance^*a*^
Tp0435	0.45	***
Tp0574	0.39	***
Tp0163	0.44	***
Tp0548	0.29	**
Tp0733	−0.04	
Tp0751	0.12	
Tp0768	0.57	***
Tp0769	0.34	***
Tp0859	0.50	***
Tp0865	0.24	**
Tp0954	0.41	***
Tp1038	0.32	***
Tp0117/TprC	0.31	***
Tp0621/TprJ.5′	0.16	~
Tp0897/TprK.5′	0.17	~
Tp1031/TprL	0.32	***

^*a*^ ^***^[0, 0.001]; ^**^(0.01, 0.05]; ^~^(0.05, 0.1], with square brackets indicating that the endpoints are included in the interval.

**Fig 4:**
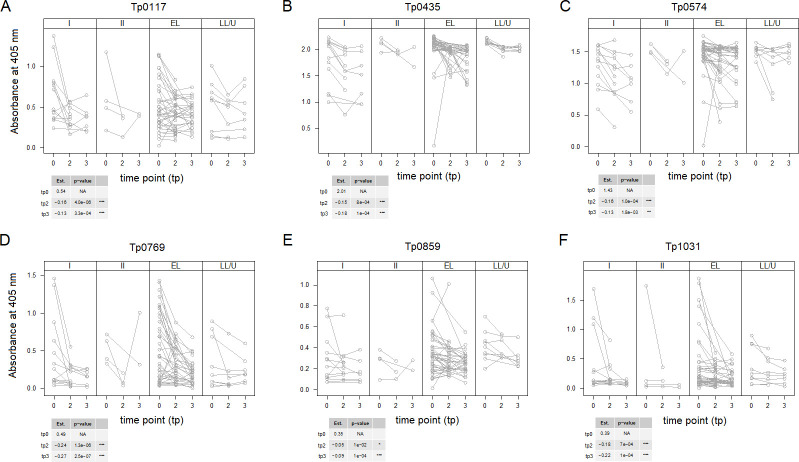
Longitudinal reactivity to selected *T. pallidum* antigens without considering covariates. Antigens to which sera reactivity decreased significantly post-treatment. Time points 0, 1, and 2 include baseline sera (tp0) and convalescent sera collected at 3 months [time point 2 (tp2)] and 6 months [time point 3 (tp3)] post-treatment. Values are displayed by stage to minimize overplotting. Antigens are reported in gene number order. In the tables associated to each figure panel, the “Est” column reports the mean reactivity for all samples at baseline (tp0 row) and the amount by which antigen reactivity declined at tp2 and tp3 compared to baseline. Asterisks indicate significance with Bonferroni-adjusted *P*-values ≤0.001 (***), ≤0.01 (**), and ≤0.05 (*). A total of 66 samples were analyzed for tp0, 51 samples for tp2, and 44 people at tp3, as not all tp3 patients provided a sample at 3 months post-treatment. The steepest declines were seen for Tp0769 (D) and Tp0117 (A).

**Fig 5:**
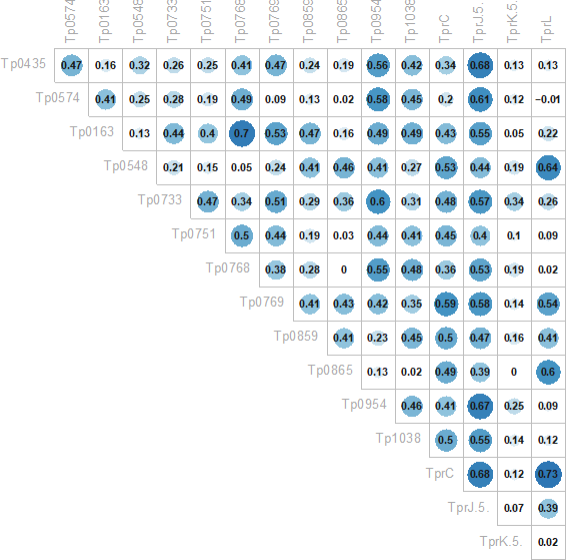
Correlation between changes in serum reactivity over time between immunodominant antigens and additional proteins in the array. Pearson correlation coefficients showing that the decrease in reactivity in Tp0769 correlates strongly with a decrease in reactivity in Tp0117/TprC, Tp0621/TprJ.5′, and Tp1031/TprL. A coefficient value ≥0.47 was used as the cut-off.

The longitudinal analysis of samples at baseline and 3 and 6 months post-treatment (without including covariates) showed a significant decrease in reactivity for six antigens (Tp0117/TprJ, Tp0435, Tp0574, Tp0769, Tp0859, and Tp1031/TprL) (Fig. 4A through F) at both time points compared to baseline, even though the steepest declines were seen in average for Tp0769 and Tp0117. *P*-values were adjusted for multiple comparisons using the Bonferroni correction. To calibrate the reduction effect, the absolute value by which antigen reactivity at time point 2 was different from baseline was divided by the range of reactivity (defined as *R* = max reactivity value − min reactivity value) for the antigen and expressed as a percent. For Tp0769, for example, the estimated amount by which antigen at time point 2 was different from baseline (−0.24) corresponded to a 16% decline of the observed range at baseline. For Tp0117, a 12% decline was observed at the 6-month post-treatment check (time point 3). Modest declines, albeit still significant, were seen for the remaining antigens, ranging from 5.1% (Tp0859) to 9.5% (for both Tp0574 and Tp1031). To further assess the credibility of these longitudinal changes in reactivity, we computed the spread of triplicate reactivity values for each sample for each antigen and then averaged the spread value across samples. A decline in reactivity from time point 2 and 3 post-treatment check was detected for all antigens but two. In the case of Tp0117 (Fig. 4A) and Tp0574 (Fig. 4C), however, the estimated amount by which antigen reactivity at time point 3 was different from baseline was slightly lower than the corresponding value for time point 2, supporting that an increase in reactivity to Tp0117 and Tp0574 might have occurred between time points 2 and 3. However, because the difference was less than the average spread of the triplicates (0.029 for Tp0117 and 0.047 for Tp0574) for these antigens at baseline, such modest increases did not appear to be meaningful. For each antigen, data were split by stage to facilitate visualization. The effect size data and calibration statistics are reported in Table S2. Furthermore, Pearson coefficient analysis using longitudinal sera pre- and post-treatment at time point 2, without including covariates, confirmed that the decrease in reactivity to Tp0769, Tp0117/TprC, and Tp0621/TprJ.5′, and Tp0131/TprL strongly correlated (Fig. 5). The descriptive statistics for changes in antigens between baseline and time point 2 are in Table S3.

**Fig 6:**
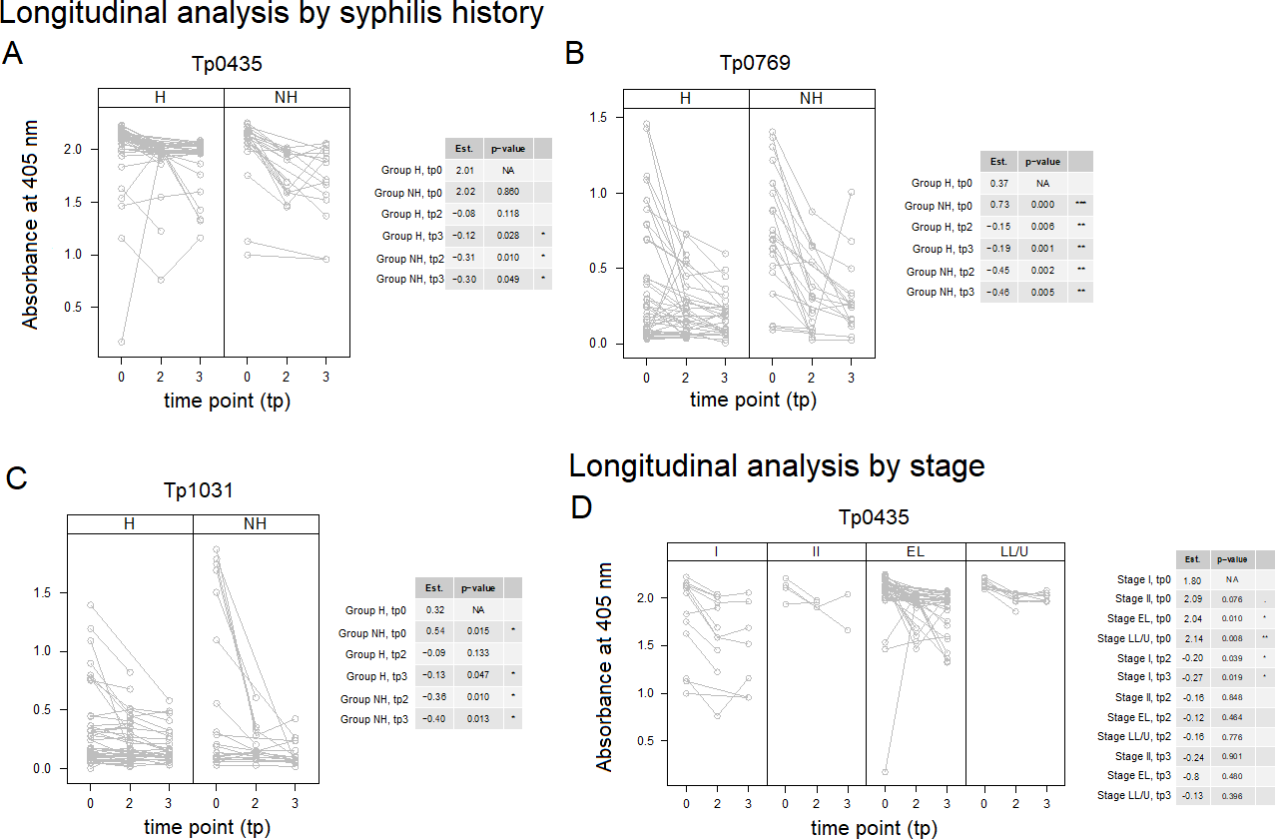
Longitudinal reactivity to selected *T. pallidum* antigens including covariates. Antigens to which sera reactivity decreased significantly post-treatment (Fig. 3) were further analyzed using syphilis history (A–C), stage (D), and HIV status. The models shown were significantly better than models using time alone. Time points 0, 1, and 2 include baseline sera (tp0) and convalescent sera collected at 3 (tp2) and 6 (tp3) months post-treatment. Antigens are reported in the gene number order. In the tables associated with each figure panel, the “Est” column reports the mean reactivity for samples in each group at baseline (tp0 row) and the amount by which antigen reactivity declined at tp2 and tp3 compared to baseline. Asterisks indicate significance with Bonferroni-adjusted *P*-values ≤0.001 (***), ≤0.01 (**), and ≤0.05 (*). No significant differences were seen when HIV status was used as a covariant. A total of 66 samples were analyzed for tp0, 51 samples for tp2, and 44 people at tp3, as not all tp3 patients provided a sample at tp2 post-treatment.

Longitudinal analysis of serum reactivity to the Tp0117, Tp0435, Tp0574, Tp0769, Tp0859, and Tp1031 antigens was repeated, including stage, HIV status (Pos or Neg), and history of infection (H or NH) as covariates. Models that were significantly better than the corresponding time-only model are shown in Fig. 6. The results (Fig. 6A through D) showed that significant differences in reactivity compared to baseline could still be seen for Tp0435, Tp0769, and Tp1031 when a history of infection was considered as a covariate (Fig. 6A through C). Although in all cases a decline in reactivity was seen at both time points 2 and 3 for all three antigens in both H and NH groups, these differences were not significant in two cases, corresponding to baseline vs tp2 in the group with a history of syphilis for Tp0435 (Fig. 6A) and the baseline vs time point 2 in the group of patients with a history of syphilis for Tp1031 (Fig. 6C). The differences in reactivity at all time points and groups for Tp0769 were highly significant (Fig. 6B). Significant differences were also found for Tp0435 when the disease stage was considered as covariate (Fig. 6D). However, a significant decline at both time points 2 and 3 compared to baseline was seen only for primary syphilis sera (Fig. 6D). Baseline reactivity of sera from early latent and late latent/unknown duration patients was also found to be significantly higher than baseline reactivity of primary sera (Fig. 6D). There were no models with HIV status that were significantly better than the time-only model for any of the above six antigens.

## DISCUSSION

Over the last two decades, the increased availability of *T. pallidum* genome sequences has provided the opportunity to produce several recombinant antigens to be tested for diagnostic use (36 – 42). These studies led to remarkable progress in achieving increased reliability of *T. pallidum*-specific serological tests based on the detection of immunodominant and highly expressed lipoproteins such as Tp0435/TpN17, Tp0574/TpN47, and Tp0171/TpN15. Indeed, many FDA-approved treponemal-specific tests use these antigens as individual recombinant proteins or chimeric concatemers in place of whole cell *T. pallidum* lysates (43), which are more difficult to attain given the complexity of propagating the syphilis spirochete either *in vivo* or *in vitro* (44, 45). Although serological assays based on recombinant lipoproteins achieved 95%–99% sensitivity and specificity, partially unsolved difficulties are related to their efficacy in identifying the early and late stages of the disease, when the humoral immunity to *T. pallidum* has yet to reach detectable levels or has decreased due to naturally occurring clearance of the pathogen by opsonophagocytosis (46), respectively. The goal of increasing the sensitivity of TTs could be attained by recruiting novel treponema antigens to add to the immunodominant lipoprotein targets. Although we did not use sera from naïve individuals and conclusions on the specificity of tested antigens cannot be drawn, our study suggested that three antigens, Tp0163 (the TroA/Tromp1 periplasmic component of the TroABC transporter) (33); Tp0954, a putative *T. pallidum* lipoprotein and placental adhesin (34); and Tp0768 (the TmpA lipoprotein) (35), correlated in reactivity to the immunodominant lipoprotein Tp0435/TpN17 and Tp0574/TpN47. This finding was not surprising for TmpA, as it was in accordance with previous studies by the van Embden group (47, 48), where this antigen was considered suitable to increase test sensitivity based on the reactivity with ~70 sera from patients at different stages of syphilis over two studies. In our study, however, serum reactivity to TmpA was significantly lower to both Tp0435/TpN17 and Tp0574/TpN47, and the spread of reactivity values across stages (Fig. 2B) was generally large, suggesting limited use of this antigen to increase the detection of early cases. Ijsselmuiden et al. (47) reported a sharp decrease in seroreactivity to TmpA 1 year post-treatment. Our data, however, could not confirm this finding when reactivity to this antigen was evaluated in sera at tp0 (pre-treatment) and time point 2 and time point 3 (3 and 6 months post-treatment, respectively). The association between the decrease in RPR titer post-treatment and the decrease in reactivity to TmpA was also not significant. Tp0954 and Tp0163 were reported to be immunogenic during infection by Brinkman et al. (31) and McGill et al. (49) but not extensively studied for their diagnostic potential. Although the overall pattern of reactivity to Tp0163 did not differ significantly from that of Tp0768 (Fig. 1B), the mean reactivity to Tp0954 was not significantly different from that of Tp0574. However, the large variability in reactivity among samples also suggests that the use of these antigens for early detection might not be appropriate. Although not desirable to perform early diagnosis, a large spread of reactivity values could pinpoint antigens that could be pursued as biomarker predictors of stage or syphilis history. Interestingly, reactivity to TmpA at baseline was found to be different in primary syphilis sera compared to samples from early latent and late latent/unknown duration patients (Fig. 2B) and patients with or without HIV infection (Fig. 2D). However, lack of significance in TmpA reactivity between primary and secondary syphilis samples and between secondary and early latent/late latent/unknown duration samples suggests that using this antigen for staging could be impractical.

Another limitation is the continued reactivity of modern conventional TTs after successful treatment that makes them unsuitable for monitoring treatment response, relapse, or reinfection in previously treated patients, a goal currently achieved using low-specificity NTTs. In this context, the identification of novel *T. pallidum* antigens, whose antibodies are rapidly eliminated from the host circulation, could lead to tests that could complement the NTTs, which would be helpful in the case of serological non-responders, who do not achieve the fourfold dilution in NTT titer to decree serological cure, or serofast patients who do not serorevert even if the proper reduction in NTT titer was achieved (50). The study of antibody kinetics to antigens recognized by the host response to a lesser extent than the immunodominant lipoproteins could offer an immunological fingerprint to achieve the differentiation of syphilis stages and assess response to treatment. In our study, although reactivity to several antigens underwent significant reduction over time, a significant association with RPR titer drop could be seen only for Tp0679/TmpB (Fig. 4D and 6B). This finding was not entirely novel, as it was previously reported by Schouls et al. (48), supporting a possible use of TmpB for monitoring serological responses regardless of HIV status (Fig. 6B), even though no statistical significance was achieved when syphilis history was considered as a covariate.

Based on the data presented here, developing a pan-proteomic array of *T. pallidum* recombinant antigens remains a priority. Work our groups are carrying on in collaboration with Dr. Joe Campo at Antigen Discovery Inc. allowed us to develop a novel and comprehensive array carrying not only the complete *T. pallidum* proteome but also antigen variants based on the Nichols and SS14 strains, which are representative of the two main circulating clades of *T. pallidum*. This array will provide a more comprehensive analysis of the sera used here as well as sera collected from experimentally infected animals over time pre- and post-treatment to better define whether any treponemal antigen or combination thereof can be suitable for immunoassays with advanced diagnostic capabilities to fulfill the tasks of facilitating the early detection of infection, differentiating syphilis stages, and confirming pharmacological cure.

### Conclusions

The minimal proteomic array that we developed and the use of a substantial number of highly characterized serum samples from syphilis patients collected pre- and post-therapy allowed us to investigate whether treponemal antigens could be used to increase the sensitivity of current assays based on recombinant antigens, allow disease staging, and monitor response to appropriate treatment. Although none of the antigens analyzed here could effectively achieve these goals with these serum samples, our results suggest that the observed changes in reactivity indicate the value of exploring additional treponemal antigens using more comprehensive proteomic arrays that can allow high-throughput parallel examination of patient sera possibly using the whole *T. pallidum* proteome.

## MATERIALS AND METHODS

### Study sites and population

Study participants were recruited between 2019 and 2021 from five sexual health clinics, three in metropolitan areas of Lima (Independencia, San Juan de Lurigancho, and Barranco districts, respectively), one in the Callao region, and one in the city of Pucallpa in eastern Peru. All centers provide testing and care for sexually transmitted infections (STIs) and are frequented by MSMs, transgender women, and sex workers. To be eligible for enrollment, participants had to be 18 or older and newly diagnosed with active syphilis, with documentation of prior syphilis testing and/or treatment at the recruitment site, availability to return to the site for follow-up visits at 3 and 6 months post-treatment, and willingness to provide the study-related biological specimens. Two groups of individuals were recruited in this study: individuals without a history of syphilis (NH) and individuals with repeat infection (H). Participants were defined as syphilis naïve if they had a newly reactive RPR at enrollment, a positive TPPA test, and a documented negative treponemal antibody test taken within the prior 12 months. Repeat syphilis infection was defined by a new fourfold titer increase or newly reactive RPR with a previously RPR test titer ≥1:8 or a documented positive treponemal antibody test. A minority group of individuals with unknown syphilis history at the recruitment site but presenting syphilitic-like genital lesions was also enrolled. Subjects meeting the eligibility criteria and willing to participate signed an informed consent form and received appropriate reimbursement for transportation costs. Cohort data are reported in Table 2. Disease staging was performed according to the CDC guidelines (https://ndc.services.cdc.gov/case-definitions/syphilis-2018/).

**TABLE 2:** Characteristics of the participants enrolled in this study

	*n*	%
Age		
18–25 yo	30	25%
26–30 yo	34	28%
31–35 yo	25	20%
>35 yo	33	27%
Sexual identity		
Cis man^*a*^	102	84%
Trans woman	16	13%
Cis woman	4	3%
Sexual worker		
No	98	80%
Yes	24	20%
HIV status		
Infected	46	38%
Uninfected	76	62%
History of previous TP infection		
Previous TP infection	60	49%
No previous TP infection	34	28%
Unknown history	28	23%
Syphilis clinical stage		
Primary	41	34%
Secondary	9	7%
Early latent	57	47%
Late latent/unknown duration	13	11%
Other^*b*^	2	2%
Cutaneous/mucosa ulcer at any place^*c*^		
No	75	61%
Yes	47	39%
Cutaneous/mucosa ulcer at anogenital region		
No	77	63%
Yes	45	37%
Cutaneous rash		
No	111	91%
Yes	11	9%

^*a*^ Only three were not MSM.

^*b*^ Suspected tertiary.

^*c*^ Includes oral lesions.

### Specimen collection and testing

At the clinic sites, serum extracted by centrifugation from venous blood was tested onsite with a rapid treponemal test (Determine Syphilis Treponema pallidum (TP), Abbott, Chicago, IL), while the RPR test (Wiener Laboratorios, Santa Fe, Argentina) was used for non-treponemal antibody screening. As previously reported, the Abbott Determine Syphilis TP had a very high sensitivity [88%; 95% confidence interval (CI), 81%–96%), the lowest rate of indeterminate tests (0.8%), and 100% specificity (51). Furthermore, in the same study, the Determine Syphilis TP showed excellent performance on fingerstick specimens, exhibiting 100% sensitivity (95% CI, 93%–100%), 100% specificity, and 2.9% indeterminate results (51). No equipment for dark-field microscopy is available at the clinic sites. Samples were also tested for HIV either with the Determine HIV-1/2 Ag/Ab Combo (Abbot) or the HIV 1/2 Ab Plus Combo Rapid Test (CTK Biotech, Poway, CA). Serum aliquots were then reserved for analysis using the antigen array described in this study. All samples were stored and shipped frozen to the Laboratory of Sexual Health at Universidad Peruana Cayetano Heredia for additional testing and long-term storage at −80°C until use. Confirmatory testing of syphilis was done using the Fujirebio (Tokyo, Japan) TPPA assay, irrespective of the RPR result attained at the clinics. Our approach for testing for syphilis was based on evidence that when using the traditional algorithm (non-treponemal test screening followed by treponemal test as confirmatory), more active syphilis cases can be identified, but some very early syphilis cases and late latent ones can be missed, as non-treponemal antibodies might be absent. If, on the contrary, the reverse algorithm is implemented (treponemal test screening followed by non-treponemal test), some subjects with previous syphilis episodes might be managed as active cases if clinical history is not available (52). The results of HIV and syphilis tests were communicated to participants along with appropriate counseling. Appropriate treatment or referral to HIV care was provided if necessary. All syphilis infections were treated on-site using antibiotics approved by the CDC STI Treatment Guidelines (53). Individuals also diagnosed with HIV were referred to the appropriate services for further evaluation of their clinical picture and care.

### *T. pallidum* antigen production and ELISA

The list of proteins included in the antigen array, their hypothetical or annotated function, sequence boundaries, and expression vectors are reported in Table 3. With two exceptions, all proteins were expressed as full-length mature antigens [i.e., lacking the predicted cleavable signal peptide, identified using PrediSi (http://www.predisi.de/) and/or SignalP 5.0 (https://services.healthtech.dtu.dk/service.php?SignalP-5.0)]. However, for two antigens, namely, TprK (Tp0897) and TprJ (To0621), only the more conserved amino-terminal portions were expressed and used for enzyme-linked immunosorbent assays (ELISAs). Coding sequences (synthesized by GenScript as codon-optimized open reading frames for expression in *E. coli*) were obtained based on the Nichols strain genome of *T. pallidum* (32) (NC_021490.2 /CP004010.2) and were cloned either into the pEXP-5-NT (Life Technologies, Carlsbad, CA) plasmid, which adds an amino-terminal His-tag, or the pET28a+/pET23b+ vectors (Millipore Sigma, St. Louis, MO) between restriction sites that would retain the vector-encoded amino (pET28a+) or carboxyl-terminal (pET23b+) His-tag, respectively. Post-cloning lack of amplification errors was assessed by Sanger sequencing. Plasmids were then transformed into *E. coli* Rosetta2 DE3 pLysS BL21 cells (Millipore Sigma), and cells were grown in auto-inducing media prepared according to Studier (54) at room temperature for 36 hours. Protein expression and solubility prior to purification were assessed by performing SDS-PAGE separation and western blot with anti-His antibodies (Millipore Sigma) as previously described (55). Proteins were purified using a nickel-based affinity chromatography system (Qiagen, Germantown, MD) under either native or denaturing conditions. Insoluble antigens were isolated following three rounds of sonication/centrifugation of culture pellets in 1× binding buffer (0.5 M NaCl, 20 mM Tris-HCl, 5 mM imidazole, pH 7.9) to eliminate soluble components. Cell pellets were then resuspended in 1× binding buffer supplemented with 6 M urea and incubated on ice for 1 hour to solubilize the target antigen. The resulting suspension was centrifuged and filtered through a 0.45-µm membrane prior to affinity chromatography as previously described (55). Soluble proteins (Tp0435, Tp0574, Tp0768, Tp0163, and Tp0751) were instead purified directly from the culture supernatant after sonication, centrifugation, and filtering. All purified antigens were dialyzed against 1× phosphate-buffered saline (PBS). Protein size and purity were assessed using SDS-PAGE, and concentration was determined via a Bicinchoninic Acid Assay kit (Fisher Scientific, Hampton, NH).

**TABLE 3:** Recombinant proteins used in this study

Protein ID	Antigen name; putative/known function	Sequence boundaries/aa positions	Expression vector/restriction sites
Tp0117	TprC; putative porin	GVLTPQ-GMKVTW/23-598	pET28a(+)/NdeI-XhoI
Tp0163	TroA; ABC transporter, periplasmic binding protein	AFGSKD-VAALAR/23-308	pEXP-NT/NA^*a*^
Tp0435	17 kDa lipoprotein; putative adhesin	CTTVCP-KKTKK/25-156	pEXP-NT/NA^*a*^
Tp0548	FadL homolog; porin transporter	LPVLA-LFDILN/33-434	pET28a(+)/NdeI-XhoI
Tp0574	47 kDa lipoprotein; cell wall biogenesis	ETHYGY-AKVVAQ/26-434	pEXP-NT/NA^*a*^
Tp0621	TprJ; putative porin	QEFSPK-GNQHQS/24-284	pET23b(+)/NdeI-XhoI
Tp0733	OmpW homolog, porin transporter	SSEGVR-GVRYHF/25-219	pET28a(+)/NdeI-XhoI
Tp0751	Pallilysin; vascular adhesin	HVPPRR-ASAPSP/29-237	pEXP-NT/NA^*a*^
Tp0768	TmpA lipoprotein	AKEEAE-EGASR/27-345	pEXP-NT/NA^*a*^
Tp0769	TmpB lipoprotein	SYDDNE-KYRPLP/25-325	pET28a(+)/NdeI-HindIII
Tp0865	FadL homolog; porin transporter	ARSFLS-DFHFLH/24-479	pET28a(+)/NdeI-XhoI
Tp0859	FadL homolog; porin transporter	HVADAP-HTRGGG/45-232	pET28a(+)/NdeI-XhoI
Tp0897	TprK, putative porin	AQVSFT–GLCALA/54-301	pET23b(+)/NdeI-XhoI
Tp0954	Lipoprotein, putative adhesin	SERAQL-SLRTNP/31-478	pEXP-NT/NA^*a*^
Tp1031	TprL, putative porin	SEQLGI-GLKIAW/26-578	pET28a(+)/NdeI-XhoI
Tp1038	TpF1 antigen (a.k.a. antigen 4D)	MNMCTD-GATLKA/1-177	pEXP-NT/NA^*a*^

^*a*^ Not applicable (the pEXP-NT vector uses TA cloning).

For ELISAs, sufficient 96-well flat bottom plates (Fisher Scientific) were prepared for each antigen to perform the assay in triplicate for each serum sample. No-antigen control wells were also included for each tested serum to evaluate background reactivity. More specifically, plates were coated with 15 pmol/well of the test antigen in 1× PBS. Once the antigen was added, the plates were incubated at 37°C for 2 hours, then refrigerated at 4°C overnight. The following day, the plates were washed with a 1 × PBS solution containing 0.05% Tween20 detergent. Four consecutive washes were performed for a total of 12 min. Subsequently, 200 µL of blocking solution made of 3% non-fat milk in 1× PBS (NFM-PBS) was applied to each well, and plates were incubated at 4°C overnight. The following day, the washing step was repeated, and after that, 100 µL of immune sera diluted 1:20 in 1% NFM-PBS was added to wells. Afterward, the plates were incubated at 4°C overnight. Subsequently, plates were washed again, and 100 µL of a secondary anti-human IgG conjugated with alkaline phosphatase (Millipore Sigma) diluted 1:2,000 in 1% NFM-PBS was added to each well. After incubation on a shaker at room temperature for 90 min, the washing step was repeated. In the meantime, a p-nitrophenyl phosphate (pNPP) developing solution was prepared by dissolving one pNPP tablet (Fisher Scientific) per 15 mL of 1× glycine buffer (0.1 M glycine buffer, pH 10.4, with 1 mM MgCl_2_ and 1 mM ZnCl_2_) prepared as per the manufacturer’s instruction, and 50 µL was added to each well. Absorbance at 405 nm was read using a Synergy HTX plate reader (BioTek Instruments Inc., Winooski, VT) 45 min after adding the pNPP solution. For each sample, the corresponding no-antigen background was subtracted to each of the three replicates. The spread of these three background-subtracted measurements (maximum − minimum) for each antigen was averaged across samples, yielding an average spread for each antigen.

### Data preparation for statistical analysis

The average of the “no antigen” wells was subtracted from the antigen wells. To calibrate the precision of the assay, for each sample for each replicate, the corresponding no-antigen background was subtracted. The spread of these three background-subtracted measurements (maximum − minimum) for each antigen was averaged across samples, yielding an average spread for each antigen. Change in antigen from baseline to time point 2 (3 months post-treatment) was calculated as time point 2 − baseline using the background-subtracted average of triplicates.

### Statistical analysis

A variety of analyses were conducted to provide insight into the clinical relevance of this panel of antigens. In each analysis, all relevant samples were included. Exclusion of data due to missing clinical measurements (e.g., RPR); nuances in covariates, such as non-informative values (e.g., unknown history); or low-frequency disease stage or manifestations (e.g., suspected tertiary or neurosyphilis) led to different sample sets for different analyses. All analysis was performed in the R statistical programming environment (56).

Pearson correlations for all antigens to each other were calculated using the R function cor and plotted using the R function corrplot. Correlation matrices were calculated for both baseline values (*n* = 120) and change in antigen (*n* = 51). Pearson correlations of baseline antigen to baseline RPR dilution (*n* = 120) were also calculated. Pearson correlations were also calculated for baseline antigen to change in RPR dilution (*n* = 64) and change in antigen to change in RPR dilution (*n* = 51).

For each antigen, Student’s *t*-tests were used to identify differences between those participants with and without a history of syphilis (*n* = 93 samples total) and between those patients with and without HIV (*n* = 120 samples total). ANOVA was used to identify differences at baseline across disease stage (primary, secondary, early latent, and late latent/unknown, *n* = 118). For all three analyses, a *P*-value <0.05 was used to identify antigens with significantly different reactivity among groups. Comparisons across the four stages were corrected for multiple comparisons using Tukey honestly significant difference (HSD) test with a significance level of *P* < 0.1.

In a primary longitudinal analysis, for each antigen, change over time was modeled with linear mixed effects (R library nlme, method lme) (57) with baseline and 3-month and 6-month time points treated as categorical variables (66 people, 161 samples). The average spread values, defined above, were used to assess the technical significance of estimated changes over time. Bonferroni adjusted *P*-values <0.05 on the change at 3 months post-treatment (time point 2) were used to identify antigens with significant changes over time.

In a subanalysis, similar longitudinal models were created using covariates. The form of the model was antigen~time + covariate + time × covariate. Separate models were created for stage, history, and HIV status. Time was treated as a categorical variable. Antigens that were significant by time only and had models with covariates that were significantly better than the models using time only (by ANOVA) were presented for discussion.

## SUPPLEMENTAL MATERIAL

Supplemental material spectrum.03466-23-s0001.docx Tables S1 to S3.

ASM does not own the copyrights to Supplemental Material that may be linked to, or accessed through, an article. The authors have granted ASM a non-exclusive, world-wide license to publish the Supplemental Material files. Please contact the corresponding author directly for reuse.
