# Supplementary material for: Evaluation of a minimal array of Treponema pallidum antigens as biomarkers for syphilis diagnosis, infection staging, and response to treatment
Source: Microbiol Spectr. 2023 Dec 14;12(1):e03466-23. doi: 10.1128/spectrum.03466-23 (PMC10782976; doi:10.1128/spectrum.03466-23)
Supplement: Supplemental material — Tables S1 to S3. [file spectrum.03466-23-s0001.docx]

**Table S1. Descriptive statistics for data in Figure 1A**

|  | Mean | StdDev | %CV | Min | Max |
| --- | --- | --- | --- | --- | --- |
| Tp0435 | 1.92 | 0.31 | 16.07 | 0.18 | 2.25 |
| Tp0574 | 1.35 | 0.30 | 22.38 | 0.02 | 1.75 |
| Tp0163 | 0.50 | 0.28 | 57.00 | 0.00 | 1.25 |
| Tp0548 | 0.83 | 0.36 | 43.74 | 0.10 | 1.88 |
| Tp0733 | 0.81 | 0.32 | 40.23 | 0.14 | 1.71 |
| Tp0751 | 0.60 | 0.34 | 57.40 | 0.07 | 2.01 |
| Tp0768 | 0.61 | 0.28 | 45.26 | -0.01 | 1.30 |
| Tp0769 | 0.39 | 0.37 | 94.55 | 0.01 | 1.69 |
| Tp0859 | 0.32 | 0.20 | 61.83 | 0.02 | 1.61 |
| Tp0865 | 0.30 | 0.33 | 110.15 | 0.03 | 1.89 |
| Tp0954 | 1.49 | 0.54 | 36.31 | 0.11 | 3.37 |
| Tp1038 | 0.22 | 0.13 | 57.23 | -0.01 | 0.72 |
| Tp0117/TprC | 0.48 | 0.26 | 54.95 | 0.03 | 1.38 |
| Tp0621/TprJ.5’ | 0.44 | 0.32 | 73.13 | 0.07 | 1.98 |
| Tp0897/TprK.5’ | 1.19 | 0.41 | 34.33 | 0.13 | 2.21 |
| Tp1031TprL | 0.29 | 0.36 | 126.38 | 0.00 | 1.87 |

%CV = (StdDev/Mean) x 100. Allows for comparison of variability across antigens. Sometimes used to characterize variability of replicates, with < 20% considered reasonable for ELISA. Because here we are looking at multiple biological samples, so we should expect CVs > 20%. Higher SDs and CVs might reflect biological differences across samples.

**Table S2. Summary of longitudinal models without covariates**

| **Antigen** | **e.tp2^1^** | **e.tp3^1^** | **p.tp2^2^** | **p.tp3^2^** | **p.tp2Bon^3^** | **p.tp3Bon^3^** | **Spread^4^** | **Range of baseline^5^** | **tp2PctRange^6^** |
| --- | --- | --- | --- | --- | --- | --- | --- | --- | --- |
| **Tp0435** | -0.15 | -0.18 | 8.49E-04 | 1.41E-04 | 1.36E-02 | 2.26E-03 | 0.031 | 2.1 | 7.3 |
| **Tp0574** | -0.16 | -0.13 | 5.56E-05 | 1.76E-03 | 8.89E-04 | 2.81E-02 | 0.047 | 1.7 | 9.5 |
| **Tp0769** | -0.24 | -0.27 | 1.28E-06 | 2.54E-07 | 2.05E-05 | 4.07E-06 | 0.030 | 1.4 | 16.7 |
| **Tp0859** | -0.05 | -0.09 | 1.01E-02 | 5.10E-05 | 1.61E-01 | 8.16E-04 | 0.018 | 1.0 | 5.1 |
| **TprC** | -0.16 | -0.13 | 4.01E-06 | 3.31E-04 | 6.41E-05 | 5.29E-03 | 0.029 | 1.4 | 12.0 |
| **TprL** | -0.18 | -0.22 | 6.58E-04 | 7.17E-05 | 1.05E-02 | 1.15E-03 | 0.013 | 1.9 | 9.5 |

^1^ Estimated amount by which antigen at tp2, tp3 is different than at baseline.

^2^ Corresponding p-values

^3^ Bonferroni-adjusted p-values

^4^ Mean value of spread of triplicates. In all cases, e.tp2 > spread

^5^ Range of antigen at baseline (max – min)

^6^ Absolute value e.tp2 /range expressed as a percent for calibrating the effect size.

Filtered models to include only those with Bonferroni corrected p-values on tp2 or tp3 < 0.05

In all cases where Bonferroni corrected p-value on tp2 < 0.05, the Bonferroni corrected p-value on tp3 was < 0.05.

**Table S3. Descriptive statistics for data in Figure 5**

|  | Mean | StdDev | %CV | Min | Max |
| --- | --- | --- | --- | --- | --- |
| Tp0435 | -0.15 | 0.34 | 227.02 | -0.70 | 1.82 |
| Tp0574 | -0.16 | 0.32 | 206.74 | -1.06 | 1.42 |
| Tp0163 | -0.09 | 0.28 | 317.55 | -0.78 | 0.74 |
| Tp0548 | -0.06 | 0.35 | 594.43 | -1.34 | 0.88 |
| Tp0733 | 0.02 | 0.26 | 1133.49 | -0.94 | 0.65 |
| Tp0751 | -0.04 | 0.20 | 533.61 | -0.60 | 0.42 |
| Tp0768 | -0.10 | 0.30 | 298.20 | -0.63 | 0.77 |
| Tp0769 | -0.25 | 0.37 | 147.65 | -1.15 | 0.69 |
| Tp0859 | -0.06 | 0.14 | 244.62 | -0.56 | 0.35 |
| Tp0865 | -0.10 | 0.30 | 296.94 | -1.38 | 0.30 |
| Tp0954 | -0.08 | 0.51 | 618.43 | -1.53 | 1.79 |
| Tp1038 | 0.00 | 0.12 | 5622.53 | -0.27 | 0.40 |
| Tp0117/TprC | -0.17 | 0.26 | 152.02 | -0.89 | 0.36 |
| Tp0621/TprJ.5’ | -0.10 | 0.31 | 317.68 | -0.78 | 1.52 |
| Tp0897/TprK.5’ | -0.16 | 0.52 | 331.24 | -1.37 | 0.92 |
| Tp1031TprL | -0.18 | 0.39 | 220.26 | -1.59 | 0.17 |

%CV = (StdDev/Mean) x 100. Allows for comparison of variability across antigens. Sometimes used to characterize variability of replicates, with < 20% considered reasonable for ELISA. Because here we are looking at multiple biological samples, so we should expect CVs > 20%. Higher CVs compared to Table S1 are driven in part by the low means.
